# Initial use of one or two antibiotics for critically ill patients with community-acquired pneumonia: impact on survival and bacterial resistance

**DOI:** 10.1186/cc13095

**Published:** 2013-11-07

**Authors:** Christophe Adrie, Carole Schwebel, Maïté Garrouste-Orgeas, Lucile Vignoud, Benjamin Planquette, Elie Azoulay, Hatem Kallel, Michael Darmon, Bertrand Souweine, Anh-Tuan Dinh-Xuan, Samir Jamali, Jean-Ralph Zahar, Jean-François Timsit

**Affiliations:** 1 Physiology Department, Paris University, Cochin Hospital 27, rue du Faubourg Saint-Jacques, Paris, France; 2 Polyvalent ICU, Delafontaine Hospital, Saint Denis, France; 3 Polyvalent ICU, University Grenoble 1, Albert Michallon Hospital, Grenoble, France; 4 ICU, Saint Joseph Hospital, Paris, France; 5 University Grenoble 1, Integrated Research Center U823, Grenoble, France; 6 Medical Surgical ICU, André Mignot Hospital, Versailles-Le Chesnay, France; 7 Medical ICU, Saint Louis Hospital, Paris, France; 8 ICU, Cayenne General Hospital, Cayenne, France; 9 Medical ICU, Saint-Etienne University Hospital, Saint-Etienne, France; 10 ICU, Gabriel Montpied Hospital, Clermont-Ferrand, France; 11 Physiology Department, Cochin Hospital, Paris, France; 12 ICU, Dourdan Hospital, Dourdan, France; 13 Microbiology Department, Necker Hospital, Paris, France

## Abstract

**Introduction:**

Several guidelines recommend initial empirical treatment with two antibiotics instead of one to decrease mortality in community-acquired pneumonia (CAP) requiring intensive-care-unit (ICU) admission. We compared the impact on 60-day mortality of using one or two antibiotics. We also compared the rates of nosocomial pneumonia and multidrug-resistant bacteria.

**Methods:**

This is an observational cohort study of 956 immunocompetent patients with CAP admitted to ICUs in France and entered into a prospective database between 1997 and 2010.

Patients with chronic obstructive pulmonary disease were excluded. Multivariate analysis adjusted for disease severity, gender, and co-morbidities was used to compare the impact on 60-day mortality of receiving adequate initial antibiotics and of receiving one versus two initial antibiotics.

**Results:**

Initial adequate antibiotic therapy was significantly associated with better survival (subdistribution hazard ratio (sHR), 0.63; 95% confidence interval (95% CI), 0.42 to 0.94; *P* = 0.02); this effect was strongest in patients with *Streptococcus pneumonia* CAP (sHR, 0.05; 95% CI, 0.005 to 0.46; *p* = 0.001) or septic shock (sHR: 0.62; 95% CI 0.38 to 1.00; *p* = 0.05). Dual therapy was associated with a higher frequency of initial adequate antibiotic therapy. However, no difference in 60-day mortality was found between monotherapy (β-lactam) and either of the two dual-therapy groups (β-lactam plus macrolide or fluoroquinolone). The rates of nosocomial pneumonia and multidrug-resistant bacteria were not significantly different across these three groups.

**Conclusions:**

Initial adequate antibiotic therapy markedly decreased 60-day mortality. Dual therapy improved the likelihood of initial adequate therapy but did not predict decreased 60-day mortality. Dual therapy did not increase the risk of nosocomial pneumonia or multidrug-resistant bacteria.

## Introduction

Community-acquired pneumonia (CAP) is among the most common severe infections in critically ill patients [1] and is associated with a high death toll. Failure to use adequate antibiotics (that is, antibiotics active *in vitro* on the causative organism) considerably increases the risk of death, particularly in patients with severe sepsis [2,3]. Consequently, the recommended antibiotic regimen for patients with CAP requiring hospital admission is either a fluoroquinolone alone or a combination of two antibiotics, including a macrolide [4-6]. Many trials suggest greater efficacy of dual therapy (usually with a β-lactam and a macrolide or fluoroquinolone) compared to monotherapy (usually with a β-lactam) [3,7-15]. Macrolides may be particularly useful, as they blunt the inflammatory response via immunomodulating effects and may exert effects on bacteria not included in their spectrum [16]. However, current recommendations are based chiefly on theoretical grounds, as opposed to high-quality studies [17-19]. Furthermore, a systematic review of randomized controlled trials found no evidence that empirical antibiotic therapy covering atypical pathogens improved survival or clinical efficacy in patients admitted for CAP [18].

Our primary objective in this observational cohort study of a prospective database was to determine whether using two initial antibiotics instead of one improved 60-day mortality in patients admitted to the ICU for CAP. We also assessed the effect on 60-day mortality of receiving adequate antibiotic therapy initially versus secondarily. Subgroup analyses were done in patients with specific organisms and in those with the most severe acute-illness syndromes. The risks of nosocomial pneumonia and multidrug-resistant (MDR) bacteria were compared in patients given one versus two antibiotics.

## Material and methods

### Inclusion and exclusion criteria

Because diagnostic coding using the International Classification of Diseases has been found unreliable in the ICU [20], we used parameters collected prospectively by data-capture software to identify the 956 patients admitted to 12 ICUs for CAP between 1996 and 2010 and included in the OutcomeRea® database (http://www.outcomerea.org). Patients were classified into three groups based on antibiotics received for at least 48 hours within the first three days after ICU admission: β-lactam alone, β-lactam plus macrolide and β-lactam plus fluoroquinolone.

We excluded patients given non-β-lactam monotherapy; patients with chronic obstructive pulmonary disease (COPD), pneumonia more than two days after ICU admission (possible ICU-acquired pneumonia), previous hospitalization, or immunodeficiency (or example, HIV infection, long-term glucocorticoid therapy, long-term hemodialysis, or cancer chemotherapy); [21] and patients who died within three days after ICU admission.

### Definitions

CAP was defined as symptoms and signs consistent with lower respiratory tract infection, new lung infiltrates by radiography or computed tomography, and infection acquired outside the hospital [4]. Patients with CAP were identified based on the ICU-admission diagnosis and microbiological findings in blood and respiratory tract specimens (sputum, bronchoalveolar lavage fluid, endotracheal aspirates or protected plugged catheter) [22], according to recently updated definitions developed by the Centers for Disease Control and the International Sepsis Consensus Conference [23]. We also took into account the results of urinary antigen tests for *Legionella pneumophila* (serotype 1) and *Streptococcus pneumoniae*.

Initial adequate antibiotic therapy was defined as one or more antibiotics active *in vitro* on the identified microorganisms or, in non-documented CAP, as treatment according to current guidelines [5], started at ICU admission and not requiring a change (secondary adjustment of antibiotic therapy) upon re-evaluation 48 hours later. Classical definitions were used for sepsis, severe sepsis and septic shock [24]. MDR bacteria were divided into four classes (methicillin-resistant *Staphylococcus aureus* (MRSA); extended-spectrum β-lactamase (ESBL)-producing *Enterobacteriacae*; non-fermenting bacteria (*Pseudomonas species, Acinetobacter* spp., and *Stenotrophomonas maltophilia*); and *Clostridium difficile*). Treatment duration was at least five days [5] but was otherwise at the discretion of the attending physician.

### Data collection

Data were collected daily by senior physicians in the participating ICUs. For each patient, the data were entered into an electronic case-report form using VIGIREA® and RHEA® data-capture software, and all case-report forms were then entered into the OutcomeRea® data warehouse (Outcomeréa, Paris, France). All codes and definitions were established prior to study initiation. For each patient, age, sex and McCabe score were recorded. Severity of illness was evaluated on the first ICU day using the Simplified Acute Physiology Score (SAPS II), Sequential Organ Failure Assessment (SOFA) score and Glasgow Coma Scale (GCS) score. Knaus scale definitions [25] were used to record pre-existing chronic organ failures, including respiratory, cardiac, hepatic, renal and immune system failures. Finally, the CURB-65 (Confusion, Urea, Respiratory rate, Blood pressure in ≥65-year-old patients) severity score was determined [26].

### Variables

Relationships with mortality and other endpoints were evaluated for the following variables: severity scores; age; sex; ICU and hospital stay lengths; co-morbidities; presence at admission of sepsis, severe sepsis or septic shock; use of invasive or noninvasive ventilation, inotropic agents, glucocorticoids or hemodialysis-hemofiltration; and recovered pathogens.

### Quality of the database

The data-capture software automatically conducted multiple checks for internal consistency of most of the variables at entry into the database. Queries generated by these checks were resolved with the source ICU before incorporation of the new data into the database. At each participating ICU, data quality was controlled by having a senior physician from another participating ICU check a 2% random sample of the study data. A one-day coding course was held annually for the study investigators and contract research organization monitors.

### Ethical issues

This study was approved by our institutional review board (CECIC Clermont-Ferrand - IRB n°5891; Ref: 2007-16), which waived the need for signed informed consent of the participants, in accordance with French legislation on non-interventional studies. However, the patients and their next of kin were asked whether they were willing to participate in the database, and none declined participation.

### Statistical analyses

The data are described as the number (%) for categorical variables and median (interquartile range) for continuous variables, unless stated otherwise. Comparisons relied on the Fisher exact test or *χ*2 test for categorical data and on the Kruskal-Wallis test for continuous data. The primary outcome was 60-day mortality and the secondary outcomes were first episodes of nosocomial pneumonia with and without MDR bacteria. Patient outcomes were censored 60 days after ICU admission. Because patients discharged alive from the hospital within the first 60 days represented an informative censor for assessing mortality, nosocomial infection and presence of MDR bacteria, a Fine-and-Gray adaptation of the Cox model was used. Results were expressed as sub-distribution hazard ratios (sHR) with their 95% confidence intervals (95% CIs).

Variables yielding *P-*values <0.20 by univariate analysis were entered into a multivariate model using backward selection, with *P* <0.05 considered significant. Glucocorticoid therapy and time to initial therapy were forced into the model. The following variables collected at ICU admission were considered for the multivariate model: gender, SAPSII, co-morbidities, severe sepsis, septic shock, invasive mechanical ventilation, glucocorticoid therapy, time to initial antibiotic therapy, hemodialysis-hemofiltration, bacteremia and pathogens with *P* <0.20 by univariate analysis. Among severity markers, the SAPSII was selected, because it had a better Akaike Information Criterion compared to CURB-65, or SOFA score and age. Continuous variables were proposed to the model in their native form if they verified the log-linearity assumption; otherwise, they were converted and entered as dummy variables. Clinically sound two-way interactions were tested. Sub-analyses were performed considering only patients with identified pathogens or with septic shock. Adjusted sHR values with their 95% CIs were calculated for each parameter estimate. *P-*values <0.05 were considered significant. Analyses were performed using the SAS 9.2 software package (SAS Institute, Cary, NC, USA).

## Results

### Patients

**Figure 1:**
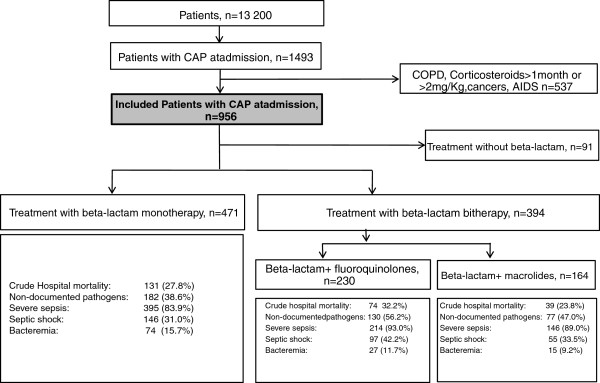
Flow diagram of the patients with community-acquired pneumonia (CAP).

Of the 13,200 patients entered into the database in 1997 to 2010, 956 met our selection criteria (see flow chart in Figure 1). Table 1 lists their main characteristics. Crude 60-day mortality was 259 (27.1%). Interestingly, the proportion of patients with *S. pneumoniae* was only 21%*,* but as many as 11% of patients had *S. aureus*, and Gram-negative bacilli were common. Of note, *Enterobacteriaceae* (all species) and *Pseudomonas aeruginosa* were found in 9% and 3% of patients, respectively.

**Table 1:** Patient characteristics and pathogens

**Parameters**	**Total (n = 956)**	Died within 60 days (n = 259, 27%)*	**Alive on day 60 (n = 697, 73%)**	***P-*** **value**
**Variables at admission**				
Male, n (%)	633 (66.2)	190 (73.4)	443 (63.6)	0.004
Age, years (median [IQR])	62 [46; 76]	72 [58; 80]	58 [42; 73]	<0.0001
**Scores, median [IQR]**				
SAPSII	43.5 [31; 59]	60 [43; 74]	39 [27; 53]	<0.0001
SOFA score	6 [4; 9]	9 [6; 12]	5 [3; 8]	<0.0001
LOD score	5 [2; 7]	7 [4; 10]	4 [2; 6]	<0.0001
Coma Glasgow Scale	13 [5; 15]	7 [3; 15]	14 [7; 15]	<0.0001
**McCabe, n (%)**				<0.0001
1	684 (71.7)	140 (54.1)	544 (78.3)	
2	235 (24.6)	98 (37.8)	137 (19.7)	
3	35 (3.7)	21 (8.1)	14 (2.0)	
**CURB-65, n (%)**				<0.0001
0	13 (1.4)	0 (0)	13 (1.9)	
1	62 (6.5)	4(1.6)	58 (8.3)	
2	176 (18.4)	22 (8.5)	154 (22.1)	
3	292 (30.6)	65 (25.2)	227 (32.6)	
4	252 (26.4)	90 (34.9)	162 (23.2)	
5	160 (16.8)	77 (29.8)	83 (11.9)	
**Co-morbidities (Knaus definitions), n (%)**				
Chronic hepatic failure	43 (4.5)	22 (8.5)	21 (3.0)	0.0003
Chronic heart failure	107 (11.2)	38 (14.7)	69 (9.9)	0.04
Chronic respiratory failure	167 (17.5)	55 (21.2)	112 (16.1)	0.06
Chronic renal failure	19 (2.0)	7 (2.7)	12 (1.7)	0.33
Diabetes	123 (12.9)	40 (15.4)	83 (11.9)	0.15
≥ One co-morbidity	352 (36.8)	124 (47.9)	228 (32.7)	<0.0001
Smokers ( >20 pack-year), n (%)	243 (30.6)	57 (26.6)	186 (32.1)	0.14
Alcohol ( >80 g/day), n (%)	189 (23.8)	44 (20.6)	145 (25.0)	0.19
Sepsis, n (%)	928 (97.1)	252(97.3)	676 (97.0)	0.80
Severe sepsis, n (%)	828 (86.6)	235 (90.7)	593 (85.1)	0.02
Septic shock, n (%)	325 (34.0)	132 (51.0)	193 (27.7)	<0.0001
**Treatments, n (%) unless otherwise stated**				
Invasive ventilation	604 (63.3)	207 (79.9)	397 (57.0)	<0.0001
Noninvasive ventilation	107 (11.2)	25 (9.7)	82 (11.8)	0.36
Inotropes or vasoactive agents	422 (44.1)	167 (64.5)	255 (36.6)	<0.0001
Corticosteroids	182 (19.0)	66 (25.5)	116 (16.6)	0.002
Hemodialysis/hemofiltration	63 (6.6)	33 (12.7)	30 (4.3)	<0.0001
Antibiotic therapy duration in days (median [IQR])	7 [4; 13]	8 [3; 15]	7 [4; 13]	0.69
**Organisms, n (%)**				
*Streptococcus pneumoniae*	202 (21.1)	52 (20.1)	150 (21.5)	0.63
*Staphylococcus aureus*	104 (10.9)	31(12.0)	73 (10.5)	0.51
*Streptococcus* spp.	36 (3.8)	12 (4.6)	24 (3.4)	0.39
*Enterocococcus* spp.	3 (0.3)	0	3 (0.4)	0.29
*Hemophilus influenzae*	97 (10.1)	25 (9.7)	72 (10.3)	0.76
*Escherichia coli*	34 (3.6)	16 (6.2)	18 (2.6)	0.008
*Enterobacter* spp.	13 (1.4)	6 (2.3)	7 (1.0)	0.12
*Klebsiella pneumoniae*	28 (2.9)	13 (5.0)	15 (2.2)	0.02
*Serratia marescens*	4 (0.4)	0 (0)	4 (0.6)	0.22
*Proteus mirabilis*	9 (0.9)	5 (1.9)	4 (0.6)	0.05
*Pseudomonas aeruginosa*	29 (3.0)	16 (6.2)	13 (1.9)	0.0005
*Legionella pneumophila*	25 (2.6)	4 (1.5)	21 (3.0)	0.21
*Mycoplasma pneumoniae*	1 (0.1)	0 (0)	1 (0.1)	0.54
*Chlamydia pneumoniae*	2 (0.2)	1 (0.4)	1 (0.1)	0.47
*Aspergillus fugimatus*	3 (0.3)	2 (0.8)	1 (0.1)	0.12
*Mycobacterium tuberculosis*	14 (1.5)	3 (1.2)	11 (1.6)	0.63
Viruses	24 (2.5)	3 (1.2)	21 (3.0)	0.10
Other	14 (1.5)	4 (1.5)	10 (1.4)	0.90
Multiple organisms	92 (9.6)	29 (11.2)	63 (9.0)	0.31
None identified	418 (43.7)	99 (38.2)	319 (45.8)	0.04
Bacteremia	121 (12.7)	45 (17.4)	76 (10.9)	0.008
**Acquisition of MDR pathogen or nosocomial pneumonia, n (%)**				
MDR bacteria	105 (11.0)	34 (13.1)	71(10.2)	0.19
MRSA	25 (2.6)	9 (3.5)	16 (2.3)	0.31
*Enterobacteriaceae* ESBL	38 (4.0)	15 (5.8)	23 (3.3)	0.08
Nonfermentative GNB	56 (5.9)	16 (6.2)	40 (5.7)	0.80
*Clostridium difficile*	9 (0.9)	2 (0.8)	7 (1.0)	0.74
Nosocomial pneumonia	127 (13.3)	48 (18.5)	79 (11.3)	0.004
ICU stay in days, median [IQR]	7 [3; 15]	8 [3; 17]	6 [3; 14]	0.45
Hospital stay in days, median [IQR]	16 [8; 31]	10 [4; 22]	18 [10; 38]	<0.0001

*273 (28.6) patients died in all, including 259 (27.1%) within 60 days and 54 (5.6%) within 48 h of ICU admission.

ESBL, extended-spectrum beta-lactamase; GNB, Gram-negative bacilli, Nonfermentative GNB (*Pseudomonas* spp.*, Acinetobacter baumannii, Stenotrophomonas maltophilia*); IQR, interquartile range; LOD, Logistic Organ Dysfunction Score; MDR, multidrug resistant; MRSA, methicillin-resistant *Staphylococcus aureus*.

As expected, survivors and non-survivors differed significantly regarding multiple organ failure prevalence, co-morbidities, gender and CURB-65 score. Compared to survivors, non-survivors had higher rates of *P. aeruginosa, Escherichia coli*, *K. pneumonia*, *P. mirabilis* and bacteremia (Table 1). However, none of these characteristics was independently associated with mortality in the multivariate analysis adjusted for confounders (SAPSII, at least one co-morbidity and gender). Nosocomial pneumonia after the CAP episode was associated with higher mortality in the unadjusted analysis but not in the adjusted analysis.

### Impact of initial adequate antibiotic therapy on 60-day mortality

Initial adequate antibiotic therapy was independently associated with better survival in the overall cohort (sHR, 0.63; 95% CI, 0.42 to 0.94.00; *P* = 0.02) (Table 2). Survival in patients who received initial inadequate therapy was not improved by secondary adjustment of the antibiotic regimen. Initial dual therapy was significantly associated with initial adequate therapy (*P* = 0.0007). There was a trend toward better survival with initial adequate antibiotic therapy in the subgroup with septic shock (sHR, 0.59; 95% CI, 0.32 to 1.08; *P* = 0.09) but not in the subgroups with sepsis or severe sepsis.

**Table 2:** Factors independently associated with 60-day mortality between initially and secondarily adequate antibiotic therapy groups

**Multivariate analysis (n = 898)**	**sHR (95% CI)**	***P-*** **value**
**Inadequate antibiotic therapy (reference)**	0.63 (0.42 to 0.94)	**0.02**
**Initial adequate antibiotic therapy**		
**Secondary adequate antibiotic therapy**	0.69 (0.37 to 1.27)	0.23
SAPSII per 10 points	1.65 (1.53 to 1.77)	<.0001
Female gender	0.70 (0.52 to 0.94)	0.02
At least one co-morbidity	1.49 (1.13 to 1.97)	0.005
Adequate antibiotic therapy on day 2 versus day 1	1.33 (0.92 to 1.93)	0.13
Adequate antibiotic therapy on day 3 versus day 1	1.29 (0.76 to 2.20)	0.35
Corticosteroids	0.98 (0.72 to 1.32)	0.87

95% CI, 95% confidence interval; SAPSII, Simplified Acute Physiology Score version II; sHR, subdistribution hazard ratio.

### Impact of one vs. two initial antibiotics on 60-day mortality

A β-lactam was used alone in 471 patients and in combination with another antibiotic in 394 patients, including 164 given a macrolide and 230 given a fluoroquinolone (Figure 1 and Table 3). The main clinical characteristics of these two groups are listed in Table 3. The fluoroquinolones were ciprofloxaxin in 56 (24%) patients, levofloxacin in 42 (18%), ofloxacin in 41 and unspecified in 38 (17%). The shorter duration of antibiotic therapy in the monotherapy group may be related to the higher rate of pathogen identification compared to the dual-therapy group. Among patients given dual therapy, those treated with fluoroquinolones had greater disease severity and a higher crude 60-day mortality rate than those given macrolides.

**Table 3:** Comparison of the groups given monotherapy and dual therapy

	**Monotherapy (β-lactam)**	**Dual therapy**		
**Parameters**	**Total population (n = 471)**	**Total population (n = 394)**	**β-lactam + Macrolide (n = 164)**	**β-lactam + Fluoroquino-lone (n = 230)**
**Variables at admission**				
Male, n (%)	301 (63.9)	274 (69.5)	110 (67.1)	164 (71.3)
Age in years, median [IQR]	60 [45; 75]	64 [48; 77]	64 [49; 79]	64 [47; 76]
**Scores, n (%)**				
SAPSII*^£^	47 [34; 60]	39.5 [28; 57]	37 [28.5; 51]	43[28; 63]
SOFA score^£^	6 [4; 9]	6 [3; 9]	5 [3; 8]	7 [4; 10]
LOD score*^£^	5 [3; 7]	4 [2; 7]	3 [2; 5.5]	4 [2; 8]
Coma Glasgow Scale*^£^	8 [4; 15]	15 [8.5; 15]	15 [11;15]	14 [7; 15]
**McCabe, n (%)**				
1	326 (69.2)	284 (72.4)	119 (73.5)	165 (71.7)
2	124 (26.3)	98 (25.0)	40 (24.7)	58 (25.2)
3	21 (4.5)	10 (2.6)	3 (1.9)	7 (3.0)
CURB-65,* n (%)				
0	3 (0.6)	7 (1.8)	1 (0.6)	6 (2.6)
1	20 (4.2)	38 (9.6)	15 (9.1)	23 (10.0)
2	88 (18.7)	71 (18.0)	34 (20.7)	37 (16.1)
3	157 (33.3)	107 (27.2)	42 (25.6)	65 (28.3)
4	122 (25.9)	107 (27.2)	46 (28.0)	61 (26.5)
5	81 (17.2)	64 (16.2)	26 (15.9)	38 (16.5)
**Co-morbidities (Knaus definitions), n (%)**				
Chronic hepatic failure^£^	23 (4.9)	17 (4.3)	3 (1.8)	14 (6.1)
Chronic heart failure	47 (10.0)	48 (12.2)	25 (15.2)	23 (10.0)
Chronic respiratory failure*^£^	65 (13.8)	86 (21.8)	52 (31.7)	34 (14.8)
Chronic renal failure	9 (1.9)	7 (1.8)	3 (1.8)	4 (1.7)
Diabetes	58 (12.3)	48 (12.2)	16 (9.8)	32 (13.9)
≥One co-morbidity*	153 (32.5)	161 (40.9)	75 (45.7)	86 (37.4)
Smokers (>20 pack-years), n (%)	114 (31.1)	97 (28.1)	41 (29.3)	56 (27.3)
Alcohol (>80 g/d)* n (%)	101 (27.6)	73 (21.2)	31 (22.1)	42 (20.5)
Sepsis, n (%)	458 (97.2)	383 (97.2)	158 (96.3)	225 (97.8)
Severe sepsis, n (%) *	395 (83.9)	360 (91.4)	146 (89)	214 (93.0)
Septic shock, n (%)*	146 (31.0)	152(38.6)	55 (33.5)	97(42.2)
**Treatments, n (%) unless otherwise stated**				
Invasive ventilation*^£^	340 (72.2)	207 (52.5)	73 (44.5	134 (58.3)
Noninvasive ventilation*	40 (8.5)	58 (14.7)	27 (16.5)	31 (13.5)
Inotropes or vasoactive agents*^£^	189 (40.1)	191 (48.5)	67 (40.9)	124 (53.9)
Corticosteroids*	69 (14.6)	96 (24.4)	40 (24.4)	56 (24.3)
Hemodialysis/Hemofil-tration	23 (4.9)	31 (7.9)	11 (637)	20 (8.7)
Antibiotic therapy duration in days, median [IQR]*	7 [4; 13]	8 [4; 14]	7 [4; 13]	8 [4; 15]
**Organisms, n (%)**				
Streptococcus pneumoniae*^£^	125 (26.5)	69 (17.5)	42 (25.6)	27 (11.7)
Staphylococcus aureus*^£^	65 (13.8)	25 (6.3)	5 (3.0)	20 (8.7)
*Streptococcus spp.*	22 (4.7)	9 (2.3)	2 (1.2)	7 (3.0)
*Enterocococcus* spp.	3 (0.6)	0 (0)	0 (0)	0 (0)
Hemophilus influenzae*	57 (12.1)	29 (7.4)	15 (9.1)	14 (6.1)
*Klebsiella pneumoniae*	16 (3.4)	8 (2.0)	3 (1.8)	5 (2.2)
*Escherichia coli*	23 (4.9)	10 (2.5)	2 (1.2)	8 (3.5)
*Enterobacteriaceae* spp.*	11 (2.3)	2 (0.5)	1 (0.6)	1 (0.4)
*Serratia marescens*	3 (0.6)	1 (0.3)	0 (0)	1 (0.4)
*Proteus mirabilis*	7 (1.5)	1 (0.3)	0 (0)	1 (0.4)
*Pseudomonas aeruginosa*	13 (2.8)	14 (3.6)	5 (3.0)	9 (3.9)
Legionella pneumophila*	1 (0.2)	9 (2.3)	5 (3.0)	4 (1.7)
*Mycoplasma pneumoniae*	0 (0)	0 (0)	0 (0)	0 (0)
*Chlamydia pneumoniae*	0 (0))	2 (0.5)	0 (0)	2 (0.9)
*Mycobacterium tuberculosis*^£^	3 (0.6)	6 (1.5)	5 (3.0)	1 (0.4)
*Aspergillus fumigatus*	0 (0)	3 (0.8)	2 (1.2)	1 (0.4)
Viruses*	6 (1.3)	15 (3.8)	7 (4.3)	8 (3.5)
Other	6 (1.3)	6 (1.5)	1 (06)	5 (2.2)
Multiple organisms*	62 (13.2)	22 (5.6)	8 (4.9)	14 (6.1)
None identified*	182 (38.6)	207 (52.5)	77 (47.0)	130 (56.5)
Bacteremia*	74 (15.7)	42 (10.7)	15 (9.1)	27 (11.7)
**Acquisition of MDR pathogens or nosocomial pneumonia, n (%)**				
MDR bacteria	52 (11.0)	44 (11.2)	19 (11.6)	25 (10.9)
MRSA	12 (2.5)	12 (3.0)	5 (3.0)	7 (3)
*Enterobacteriaceae* ESBL	22 (4.7)	12 (3.0)	5 (3.0)	7 (3)
Nonfermentative GNB	25 (5.3)	25 (6.3)	14 (8.5)	11 (4.8)
*Clostridium difficile*	6 (1.3)	2 (0.5)	0 (0)	2 (0.9)
Nosocomial pneumonia	58 (12.3)	54 (13.7)	23 (14)	31 (13.5)
**ICU stay in days, median [IQR]**	6 [3; 15]	7 [3; 16]	7 [3.5; 15.5]	8 [3; 17]
**Hospital stay in days, median [IQR]**	15 [8; 33]	18 [10; 31]	17.5 [10.5; 35]	18 [9; 30]
**Patients who died within 60 days,**^**£**^**n (%)**	123 (26.1)	107 (27.2)	35 (21.3)	72 (31.3)

ESBL, extended-spectrum beta-lactamase; LOD, Logistic Organ Dysfunction Score; MDR, multidrug resistant; MRSA, methicillin-resistant *Staphylococcus aureus*; Nonfermentative GNB, nonfermentative Gram-negative bacilli (*Pseudomonas* spp.*, Acinetobacter baumannii, Stenotrophomonas maltophilia*).

*P <0.05 for monotherapy (n = 471) versus dual therapy (n = 394); ^£^*P* <0.05 for β-lactam + macrolide (n = 164) vs. β-lactam + fluoroquinolone (n = 230).

By multivariate analysis, 60-day mortality was not significantly different between dual therapy and monotherapy (sHR, 1.14; 95% CI, 0.86 to 1.50; *P* = 0.37), even when the analysis was restricted to patients with *S. pneumonia,* those with documented infection, or those with septic shock (Table 4). By multivariate analysis, 60-day mortality was not significantly different between the macrolide and fluoroquinolone subgroups (sHR, 1.45; 95% CI, 0.78 to 2.70; *P* = 0.24), even when the analysis was restricted to patients with *S. pneumonia,* those with documented infection, or those with septic shock. These results were unchanged when the analysis was confined to patients given initial adequate antibiotic therapy.

**Table 4:** Factors associated with 60-day mortality in the groups given monotherapy or dual therapy

**Multivariate analysis**	**sHR**	**95% CI**	***P-*** **value**
Dual therapy versus monotherapy	1.14	0.86 to 1.50	0.37
SAPSII (per 10 points)	1.66	1.54 to 1.79	<.0001
Female gender	0.72	0.53 to 0.96	0.03
≥One co-morbidity	1.43	1.07 to 1.91	0.01
Antibiotic therapy on Day 2 versus Day 1	1.38	0.98 to 1.93	0.07
Antibiotic therapy on Day 3 versus Day 1	1.28	0.75 to 2.19	0.36
Steroids	0.97	0.71 to 1.32	0.83
Multivariate sensitivity analysis*	**sHR**	**(95% CI)**	***P*-value**
Restricted to *Streptococcus pneumoniae* infection	1.42	0.73 to 2.77	0.31
Restricted to documented infection	1.29	0.89 to 1.89	0.18
Restricted to patients with septic shock	1.11	0.75 to 1.64	0.59

*Adjusted on SAPSII, female gender, at least one co-morbidity, day of antibiotic therapy initiation and use of steroids.

The following variables at admission were considered for entry into the model: gender, SAPSII, at least one co-morbidity, severe sepsis, septic shock, invasive ventilation, steroid therapy, day of antibiotic therapy initiation, steroids, hemodialysis-hemofiltration, bacteremia and pathogens yielding *P-*values <0.2 by univariate analysis.

### Impact of one vs. two antibiotics on nosocomial pneumonia and multidrug-resistant bacteria rates

Nosocomial pneumonia developed in 127 patients, and MDR bacteria were identified in 105 patients. Neither the rate of nosocomial pneumonia nor the rate of MDR bacteria recovery differed significantly across the three antibiotic treatment groups (monotherapy, dual therapy with a macrolide and dual therapy with a fluoroquinolone; Table 3).

## Discussion

In a very large cohort of immunocompetent ICU patients with CAP, initial adequate antibiotic therapy improved 60-day survival and the improvement was greatest in the patients with *S. pneumoniae* infection or septic shock. Initial dual antibiotic therapy (β-lactam plus macrolide or fluoroquinolone) was associated with a higher frequency of adequate initial therapy but was not associated with better 60-day survival compared to β-lactam monotherapy. Dual therapy did not significantly affect the risk of nosocomial pneumonia or MDR bacteria compared to monotherapy.

Our study provides a good picture of the pathogens responsible for CAP requiring ICU admission. The distribution of the pathogens was consistent with previously published results, which vary widely, however, perhaps in relation to variations in case-mix [6]. Thus, the prevalence of *S. pneumoniae* infection in our study was only about half that found in a vast cohort of 3,523 patients in Spain [27], whereas the prevalence of Gram-negative bacilli was similar. The Spanish study included 15% of outpatients and 85% of inpatients admitted to wards or ICUs, whereas we studied only ICU patients, a population possibly characterized by greater bacterial virulence, greater bacterial resistance, and/or a longer time to treatment. Despite our restrictive inclusion criteria, *Enterobacteriaceae* and *Pseudomonas aeruginosa* were identified in 9.2% and 3% of patients, respectively. These pathogens, which raise specific treatment challenges, are typically found in healthcare-associated pneumonia [28]. However, 60-day mortality was independently related to acute illness severity, acute organ dysfunction, the presence of at least one co-morbidity and gender [29], but not to the nature of the pathogens, confirming our previous findings [30].

The better 60-day survival associated with initial adequate antibiotic therapy, particularly in the patients with the greatest disease severity, is consistent with many studies documenting the importance of very early adequate antibiotic administration in septic shock and severe pneumonia [2,31,32]. Dual therapy significantly increased the frequency of initial adequate therapy but did not increase survival. This apparent inconsistency in our findings may be ascribable to the fact that adequate therapy in patients without bacteriological documentation was defined in our study as compliance with guidelines. Another possible explanation is insufficient statistical power, although our cohort was large. So the effect of dual therapy in improving initial treatment adequacy may have been too small to induce a significant decrease in mortality.

Our finding that 60-day mortality was not significantly decreased by using two initial antibiotics instead of one, even in patients with septic shock or *S. pneumoniae* infection, may appear to contradict earlier studies [3,8,9,21]. These discrepancies may be ascribable to differences in disease severity, causative organisms, antibiotics used, exclusion criteria and primary outcome measures. Thus, a prospective observational study of ICU patients found that dual therapy improved survival in the subgroup with shock, whereas no difference with monotherapy was noted in the subgroup without shock; however, survival was recorded at ICU discharge or on Day 28 [3]. In another prospective observational ICU study, in which 75.7% of the patients had shock; dual therapy, including a macrolide, was associated with better ICU survival compared to dual therapy with a fluoroquinolone [8], whereas we found no significant difference in 60-day mortality between these two groups. Although one of these studies excluded patients with COPD [3] and the other excluded patients with immunodeficiencies [8], neither used all the exclusion criteria used in our study. Two other studies focused on *S. pneumoniae* disease. One was a prospective observational study of patients with *S. pneumoniae* bacteremia showing that dual therapy improved 14-day survival in the subgroup requiring ICU admission but not in the subgroup managed on the wards [9]. In patients with *S. pneumoniae* CAP and bacteremia admitted to wards or ICUs, initial dual therapy with a macrolide was associated with better hospital survival compared to a β-lactam alone. Again, these studies had a different case-mix, as only 21.1% of our patients had *S. pneumoniae* infection and only 12.7% had bacteremia. Two studies found no difference between one and two initial antibiotics in patients with severe CAP [13,14]. One was a *post hoc* analysis of data from two clinical trials of patients with severe pneumococcal sepsis and used ICU survival as the primary outcome, [13] whereas the other was a randomized trial comparing lefloxacin alone to ofloxacin plus cefotaxime in patients with severe CAP but with no shock and used clinical efficacy as the primary outcome [14]. Thus, neither study is closely similar to ours. A review article published in 2011 [7] showed that many studies supporting initial dual therapy in the most severe CAP requiring hospital admission were often retrospective, used a broad range of antibiotic regimens with some leading to conflicting results. As a result, the applicability of their results to everyday ICU practice can be challenged. *In vitro* resistance to macrolides has been associated with a higher clinical failure rate [33]. Despite consistent reports of increasing resistance to many antibiotics [33,34], treatment failure remains extremely rare in patients with CAP. That β-lactams are not effective against *L. pneumophila* may seem to support dual therapy. However, *L. pneumophila* infection is rare and routinely sought by cultures or urine antigen testing (for type 1), with specific treatment being started at the slightest doubt, as this strategy has been shown to improve survival [35].

The main rationale for dual therapy is that the broader spectrum thus obtained covers atypical pathogens (other than *Legionella pneumophila*). However, only low-level evidence is available to support this rationale [19], as most studies were retrospective and had limited statistical power. The role for atypical pathogens in CAP has been extensively reviewed [5-7,17,18]. The absence of benefits from dual therapy on survival in our study may be partly due to the low frequency of atypical pathogens. A 2012 Cochrane review of randomized controlled trials in patients admitted to wards or ICUs for CAP found no benefit of atypical-pathogen coverage on clinical efficacy (the primary outcome) or survival, even in the subgroup of patients with atypical bacteria [36].

Another theoretical reason for using dual therapy is the possibility of improved effectiveness in patients with CAP due to resistant bacteria. We excluded patients with COPD and those admitted from long-term healthcare facilities, in whom repeated exposure to antibiotics increases the risk of bacterial resistance. The resulting low proportion of patients with initial bacterial resistance may have contributed to the absence of an effect of dual therapy on survival in our study.

Dual therapy might be expected to improve outcomes of patients with the most severe forms of CAP. Dual therapy was associated with better 28-day survival in patients with CAP and septic shock [3] and with better ICU survival in patients with severe CAP including 75.7% with septic shock [8]. In a retrospective study, treatment with a β-lactam and macrolide combination provided better 14-day and 30-day survival than did a fluoroquinolone alone in patients admitted for severe CAP [37]. In our study confined to ICU patients, dual therapy was not better than monotherapy, even when the second antibiotic was a macrolide. Patients with severe CAP usually undergo comprehensive investigations on an emergency basis, and their antibiotics are very quickly adapted to the microscopic smear results than to the susceptibility test results. This approach may decrease the impact of initial empirical dual antibiotic therapy.

Overuse of antibiotics, particularly fluoroquinolones [38,39], might increase the risks of MDR bacteria selection and nosocomial pneumonia, although convincing clinical data to support these possibilities is lacking [40]. Secondary identification of MDR bacteria and nosocomial pneumonia occurred in similar proportions of patients in the monotherapy and dual therapy groups in our study. This finding should not be construed as evidence that unnecessary antibiotic use is harmless. Widespread antibiotic overuse does select MDR bacteria. Furthermore, both macrolides and fluoroquinolones can cause arrhythmias by prolonging the QT interval [41].

The strengths of our study are the large sample size, prospective data collection, patient identification based on clinical variables as opposed to codes [20], high quality of the database, and careful adjustment for confounding variables. Furthermore, to avoid including patients with healthcare-associated infections, which require specific treatment approaches, we excluded patients with immunodeficiencies, COPD or chronic dialysis as well as patients admitted from long-term healthcare facilities. The main limitation of our study is the observational design without random allocation of the initial antibiotic regimen. Randomized studies specifically evaluating β-lactam therapy alone or with a macrolide or fluoroquinolone would probably require very large sample sizes. Also, we had no information on antibiotic use in the six months preceding the CAP episode or on recent and/or repeated contact of the patients with healthcare professionals. This last factor may explain the high proportion of patients with gram-negative bacilli in our study. However, the possibility that patients with healthcare-associated pneumonia may be at increased risk of death because of their distinctive bacteriological features (including increased resistance) was challenged recently [28]. The higher mortality in these patients may be related instead to other factors, including functional impairments, malnutrition and a more restrictive ICU-admission policy. After careful adjustment for these factors, mortality was not significantly increased. Thus, there may be no sound rationale for modifying current guidelines for this particular population [28].

## Conclusions

In conclusion, initial adequate therapy was associated with better 60-day survival in our patients with CAP requiring ICU admission and without COPD or immunodeficiency. Dual therapy (β-lactam plus macrolide or fluoroquinolone), compared to monotherapy (β-lactam alone), was associated with adequacy of initial antibiotic therapy but did not improve 60-day survival. Dual therapy did not increase the risks of nosocomial pneumonia or secondary bacterial multidrug resistance.

## Key messages

● Initial adequate therapy was associated with better 60-day survival in ICU patients admitted for community-acquired pneumonia.

● Dual therapy with a β-lactam plus a macrolide or fluoroquinolone, although usually recommended as better than β-lactam monotherapy in severe community-acquired pneumonia, improved the adequacy of initial antibiotic therapy but did not improve 60-day survival.

● Dual therapy with a β-lactam plus a macrolide or fluoroquinolone did not increase the risk of nosocomial pneumonia or multidrug-resistant bacteria.

## Abbreviations

95% CI: 95% confidence interval; CAP: Community-acquired pneumonia; COPD: Chronic obstructive pulmonary disease; CURB-65: Confusion/Urea/Respiratory rate/Blood pressure score for individuals aged 65 years or over; ESBL: Extended-spectrum beta-lactamase; GCS: Glasgow coma scale; ICU: Intensive care unit; LOD: Logistic organ dysfunction score; MDR: Multidrug resistant; MRSA: Methicillin-resistant *Staphylococcus aureus*; SAPSII: Simplified acute physiology score version II; sHR: Subdistribution hazard ratio; SOFA: Sequential organ failure assessment.

## Competing interests

The authors declare that they have no competing interests.

## Authors’ contributions

CA, LV and JFT had full access to all the data in the study and take responsibility for the integrity of the data and the accuracy of the data analysis, including, and especially, any adverse effects. CA and JFT participated in the study concept and design, the acquisition of date, the interpretation of the data, and the drafting of the manuscript. LV participated in the statistical analysis and critical revision of the manuscript. CS, MGO, BP, EA, HK, MD, BS, ATDX, SJ and JRZ made substantial contributions to conception and design, acquisition of data, or analysis and interpretation of the data; drafting the article or revising it critically for important intellectual content; and final approval of the version to be published. The final version of the manuscript was read and approved by all of the authors.

## References

[B1] Adrie C Alberti C Chaix-Couturier C Azoulay E De Lassence A Cohen Y Meshaka P Cheval C Thuong M Troché G Garrouste-Orgeas M Timsit JF Epidemiology and economic evaluation of severe sepsis in France: age, severity, infection site, and place of acquisition (community, hospital, or intensive care unit) as determinants of workload and cost J Crit Care 2005 17 46 58 10.1016/j.jcrc.2004.10.005 16015516

[B2] Kumar A Ellis P Arabi Y Roberts D Light B Parrillo JE Dodek P Wood G Kumar A Simon D Peters C Ahsan M Chateau D Cooperative Antimicrobial Therapy of Septic Shock Database Research Group Initiation of inappropriate antimicrobial therapy results in a fivefold reduction of survival in human septic shock Chest 2009 17 1237 1248 10.1378/chest.09-0087 19696123

[B3] Rodríguez A Mendia A Sirvent JM Barcenilla F de la Torre-Prados MV Solé-Violán J Rello J CAPUCI Study Group Combination antibiotic therapy improves survival in patients with community-acquired pneumonia and shock Crit Care Med 2007 17 1493 1498 10.1097/01.CCM.0000266755.75844.05 17452932

[B4] Lim WS Baudouin SV George RC Hill AT Jamieson C Le Jeune I Macfarlane JT Read RC Roberts HJ Levy ML Wani M Woodhead MA Pneumonia Guidelines Committee of the BTS Standards of Care Committee BTS guidelines for the management of community acquired pneumonia in adults: update 2009 Thorax 2009 17 iii1 iii55 19783532 10.1136/thx.2009.121434

[B5] Mandell LA Wunderink RG Anzueto A Bartlett JG Campbell GD Dean NC Dowell SF File TM Jr Musher DM Niederman MS Torres A Whitney CG Infectious Diseases Society of America; American Thoracic Society Infectious Diseases Society of America/American Thoracic Society consensus guidelines on the management of community-acquired pneumonia in adults Clin Infect Dis 2007 17 S27 S72 10.1086/511159 17278083 PMC7107997

[B6] Woodhead M Blasi F Ewig S Garau J Huchon G Ieven M Ortqvist A Schaberg T Torres A van der Heijden G Read R Verheij TJ Joint Taskforce of the European Respiratory Society and European Society for Clinical Microbiology and Infectious Diseases Guidelines for the management of adult lower respiratory tract infections–full version Clin Microbiol Infect 2011 17 E1 E59 21951385 10.1111/j.1469-0691.2011.03672.x PMC7128977

[B7] Caballero J Rello J Combination antibiotic therapy for community-acquired pneumonia Ann Intensive Care 2011 17 48 10.1186/2110-5820-1-48 22113077 PMC3248869

[B8] Martin-Loeches I Lisboa T Rodriguez A Putensen C Annane D Garnacho-Montero J Restrepo MI Rello J Combination antibiotic therapy with macrolides improves survival in intubated patients with community-acquired pneumonia Intensive Care Med 2010 17 612 620 10.1007/s00134-009-1730-y 19953222

[B9] Baddour LM Yu VL Klugman KP Feldman C Ortqvist A Rello J Morris AJ Luna CM Snydman DR Ko WC Chedid MB Hui DS Andremont A Chiou CC International Pneumococcal Study Group Combination antibiotic therapy lowers mortality among severely ill patients with pneumococcal bacteremia Am J Respir Crit Care Med 2004 17 440 444 10.1164/rccm.200311-1578OC 15184200

[B10] Mortensen EM Restrepo MI Anzueto A Pugh J The impact of empiric antimicrobial therapy with a beta-lactam and fluoroquinolone on mortality for patients hospitalized with severe pneumonia Crit Care 2005 17 R8 16420641 10.1186/cc3934 PMC1550860

[B11] Burgess DS Lewis JS 2nd Effect of macrolides as part of initial empiric therapy on medical outcomes for hospitalized patients with community-acquired pneumonia Clin Ther 2000 17 872 878 10.1016/S0149-2918(00)80059-4 10945513

[B12] Dwyer R Ortqvist A Aufwerber E Henriques-Normark B Marrie TJ Mufson MA Torres A Woodhead MA Alenius M Kalin M Addition of a macrolide to a SS-lactam in bacteremic pneumococcal pneumonia Eur J Clin Microbiol Infect Dis 2006 17 518 521 10.1007/s10096-006-0183-2 16896822

[B13] Harbarth S Garbino J Pugin J Romand JA Pittet D Lack of effect of combination antibiotic therapy on mortality in patients with pneumococcal sepsis Eur J Clin Microbiol Infect Dis 2005 17 688 690 10.1007/s10096-005-0018-6 16215712

[B14] Leroy O Saux P Bédos JP Caulin E Comparison of levofloxacin and cefotaxime combined with ofloxacin for ICU patients with community-acquired pneumonia who do not require vasopressors Chest 2005 17 172 183 10.1378/chest.128.1.172 16002932

[B15] Rello J Lisboa T Lujan M Gallego M Kee C Kay I Lopez D Waterer GW DNA-Neumococo Study Group Severity of pneumococcal pneumonia associated with genomic bacterial load Chest 2009 17 832 840 10.1378/chest.09-0258 19433527

[B16] Kanoh S Rubin BK Mechanisms of action and clinical application of macrolides as immunomodulatory medications Clin Microbiol Rev 2010 17 590 615 10.1128/CMR.00078-09 20610825 PMC2901655

[B17] Martinez FJ Monotherapy versus dual therapy for community-acquired pneumonia in hospitalized patients Clin Infect Dis 2004 17 S328 S340 10.1086/382689 15127366

[B18] Shefet D Robenshtok E Paul M Leibovici L Empirical atypical coverage for inpatients with community-acquired pneumonia: systematic review of randomized controlled trials Arch Intern Med 2005 17 1992 2000 10.1001/archinte.165.17.1992 16186469

[B19] Keeley D Guidelines for managing community acquired pneumonia in adults BMJ 2002 17 436 437 10.1136/bmj.324.7335.436 11859031 PMC1122378

[B20] Misset B Nakache D Vesin A Darmon M Garrouste-Orgeas M Mourvillier B Adrie C Pease S de Beauregard MA Goldgran-Toledano D Métais E Timsit JF Outcomerea Database Investigators Reliability of diagnostic coding in intensive care patients Crit Care 2008 17 R95 10.1186/cc6969 18664267 PMC2575581

[B21] Kollef MH Micek ST Patients hospitalized with pneumonia: determining the need for broad-spectrum antibiotic therapy Clin Infect Dis 2012 17 479 482 10.1093/cid/cir848 22109952

[B22] Pham LH Brun-Buisson C Legrand P Rauss A Verra F Brochard L Lemaire F Diagnosis of nosocomial pneumonia in mechanically ventilated patients. Comparison of a plugged telescoping catheter with the protected specimen brush Am Rev Respir Dis 1991 17 1055 1061 10.1164/ajrccm/143.5_Pt_1.1055 2024814

[B23] Calandra T Cohen J International Sepsis Forum Definition of Infection in the ICU Consensus Conference The International Sepsis Forum Consensus Conference on Definitions of Infection in the Intensive Care Unit Crit Care Med 2005 17 1538 1548 10.1097/01.CCM.0000168253.91200.83 16003060

[B24] American College of Chest Physicians/Society of Critical Care Medicine Consensus Conference: definitions for sepsis and organ failure and guidelines for the use of innovative therapies in sepsis Crit Care Med 1995 17 864 874 1597042

[B25] Knaus WA Draper EA Wagner DP Zimmerman JE APACHE II: a severity of disease classification system Crit Care Med 1985 17 818 829 10.1097/00003246-198510000-00009 3928249

[B26] Lim WS van der Eerden MM Laing R Boersma WG Karalus N Town GI Lewis SA Macfarlane JT Defining community acquired pneumonia severity on presentation to hospital: an international derivation and validation study Thorax 2003 17 377 382 10.1136/thorax.58.5.377 12728155 PMC1746657

[B27] Cillóniz C Ewig S Polverino E Marcos MA Esquinas C Gabarrús A Mensa J Torres A Microbial aetiology of community-acquired pneumonia and its relation to severity Thorax 2011 17 340 346 10.1136/thx.2010.143982 21257985

[B28] Ewig S Welte T Chastre J Torres A Rethinking the concepts of community-acquired and health-care-associated pneumonia Lancet Infect Dis 2010 17 279 287 10.1016/S1473-3099(10)70032-3 20334851

[B29] Adrie C Azoulay E Francais A Clec’h C Darques L Schwebel C Nakache D Jamali S Goldgran-Toledano D Garrouste-Orgeas M Timsit JF OutcomeRea Study Group Influence of gender on the outcome of severe sepsis: a reappraisal Chest 2007 17 1786 1793 10.1378/chest.07-0420 17890473

[B30] Zahar JR Timsit JF Garrouste-Orgeas M Francais A Vesim A Descorps-Declere A Dubois Y Souweine B Haouache H Goldgran-Toledano D Allaouchiche B Azoulay E Adrie C Outcomes in severe sepsis and patients with septic shock: pathogen species and infection sites are not associated with mortality Crit Care Med 2011 17 1886 1895 Erratum in: *Crit Care Med* 2011, **39**:2392. Vesim, Aurélien [corrected to Vesin, Aurélien] 10.1097/CCM.0b013e31821b827c 21516036

[B31] Kumar A Roberts D Wood KE Light B Parrillo JE Sharma S Suppes R Feinstein D Zanotti S Taiberg L Gurka D Kumar A Cheang M Duration of hypotension before initiation of effective antimicrobial therapy is the critical determinant of survival in human septic shock Crit Care Med 2006 17 1589 1596 10.1097/01.CCM.0000217961.75225.E9 16625125

[B32] Meehan TP Fine MJ Krumholz HM Scinto JD Galusha DH Mockalis JT Weber GF Petrillo MK Houck PM Fine JM Quality of care, process, and outcomes in elderly patients with pneumonia JAMA 1997 17 2080 2084 10.1001/jama.1997.03550230056037 9403422

[B33] Jacobs MR In vivo veritas: in vitro macrolide resistance in systemic Streptococcus pneumoniae infections does result in clinical failure Clin Infect Dis 2002 17 565 569 10.1086/341980 12173130

[B34] Weiss K Tillotson GS The controversy of combination vs monotherapy in the treatment of hospitalized community-acquired pneumonia Chest 2005 17 940 946 10.1378/chest.128.2.940 16100190

[B35] Alvarez J Dominguez A Sabria M Ruiz L Torner N Cayla J Barrabeig I Sala MR Godoy P Camps N Minguell S Impact of the Legionella urinary antigen test on epidemiological trends in community outbreaks of legionellosis in Catalonia, Spain, 1990–2004 Int J Infect Dis 2009 17 e365 e370 10.1016/j.ijid.2009.01.004 19356959

[B36] Eliakim-Raz N Robenshtok E Shefet D Gafter-Gvili A Vidal L Paul M Leibovici L Empiric antibiotic coverage of atypical pathogens for community-acquired pneumonia in hospitalized adults Cochrane Database Syst Rev 2012 17 CD004418

[B37] Lodise TP Kwa A Cosler L Gupta R Smith RP Comparison of beta-lactam and macrolide combination therapy versus fluoroquinolone monotherapy in hospitalized Veterans Affairs patients with community-acquired pneumonia Antimicrob Agents Chemother 2007 17 3977 3982 10.1128/AAC.00006-07 17709460 PMC2151470

[B38] Bliziotis IA Michalopoulos A Kasiakou SK Samonis G Christodoulou C Chrysanthopoulou S Falagas ME Ciprofloxacin vs an aminoglycoside in combination with a beta-lactam for the treatment of febrile neutropenia: a meta-analysis of randomized controlled trials Mayo Clin Proc 2005 17 1146 1156 10.4065/80.9.1146 16178494

[B39] MacDougall C Powell JP Johnson CK Edmond MB Polk RE Hospital and community fluoroquinolone use and resistance in Staphylococcus aureus and Escherichia coli in 17 US hospitals Clin Infect Dis 2005 17 435 440 10.1086/432056 16028149

[B40] Tamma PD Cosgrove SE Maragakis LL Combination therapy for treatment of infections with gram-negative bacteria Clin Microbiol Rev 2012 17 450 470 10.1128/CMR.05041-11 22763634 PMC3416487

[B41] Zambon A Polo Friz H Contiero P Corrao G Effect of macrolide and fluoroquinolone antibacterials on the risk of ventricular arrhythmia and cardiac arrest: an observational study in Italy using case-control, case-crossover and case-time-control designs Drug Saf 2009 17 159 167 10.2165/00002018-200932020-00008 19236122

